# The homeostatic function of Regnase‐2 restricts neuroinflammation

**DOI:** 10.1096/fj.202201978R

**Published:** 2023-02-08

**Authors:** Weronika Sowinska, Mateusz Wawro, Debolina D. Biswas, Jakub Kochan, Katarzyna Pustelny, Aleksandra Solecka, Angela S. Gupta, Karli Mockenhaupt, Jarosław Polak, Borys Kwinta, Tomasz Kordula, Aneta Kasza

**Affiliations:** ^1^ Department of Cell Biochemistry, Faculty of Biochemistry, Biophysics and Biotechnology Jagiellonian University Kraków Poland; ^2^ Department of Biochemistry and Molecular Biology, School of Medicine and the Massey Cancer Center Virginia Commonwealth University Richmond Virginia USA; ^3^ Department of Neurosurgery and Neurotraumatology Jagiellonian University Medical College Kraków Poland

**Keywords:** glioblastoma, neuroinflammation, proliferation, transcripts turnover

## Abstract

The precise physiological functions and mechanisms regulating RNase Regnase‐2 (Reg‐2/ZC3H12B/MCPIP2) activity remain enigmatic. We found that Reg‐2 actively modulates neuroinflammation in nontransformed cells, including primary astrocytes. Downregulation of Reg‐2 in these cells results in increased mRNA levels of proinflammatory cytokines IL‐1β and IL‐6. In primary astrocytes, Reg‐2 also regulates the mRNA level of Regnase‐1 (*Reg‐1*/ZC3H12A/MCPIP1). *Reg‐2* is expressed at high levels in the healthy brain, but its expression is reduced during neuroinflammation as well as glioblastoma progression. This process is associated with the upregulation of *Reg‐1*. Conversely, overexpression of Reg‐2 is accompanied by the downregulation of *Reg‐1* in glioma cells in a nucleolytic NYN/PIN domain‐dependent manner. Interestingly, low levels of *Reg‐2* and high levels of *Reg‐1* correlate with poor‐glioblastoma patients' prognoses. While *Reg‐2* restricts the basal levels of proinflammatory cytokines in resting astrocytes, its expression is reduced in IL‐1β‐activated astrocytes. Following IL‐1β exposure, Reg‐2 is phosphorylated, ubiquitinated, and degraded by proteasomes. Simultaneously, the *Reg‐2* transcript is destabilized by tristetraprolin (TTP) and *Reg‐1* through the AREs elements and conservative stem‐loop structure present in its 3′UTR. Thus, the peer‐control loop, of Reg‐1 and Reg‐2 opposing each other, exists. The involvement of TTP in *Reg‐2* mRNA turnover is confirmed by the observation that high TTP levels correlate with the downregulation of the *Reg‐2* expression in high‐grade human gliomas. Additionally, obtained results reveal the importance of Reg‐2 in inhibiting human and mouse glioma cell proliferation. Our current studies identify Reg‐2 as a critical regulator of homeostasis in the brain.

**Abbreviations**
ARE: adenine‐ and uridine‐rich elements
AUF‐1: AU‐binding Factor 1
BBB: brain–blood barrier
CNS: central nervous system
CRISPRi: CRISPR interference
CXCL: C‐X‐C motif chemokine ligand
DAMPS: damage‐associated molecular patterns
DDB1: DNA damage‐binding protein 1
EAE: experimental autoimmune encephalomyelitis
ERK: extracellular signal‐regulated protein kinase
GBM: glioblastoma multiforme
GM‐CSF: granulocyte‐macrophage colony‐stimulating factor
HuR: human antigen R
IL: interleukin
iNOS: inducible nitric oxide synthase
KSRP: KH‐type splicing regulatory protein
LCN2: Lipocalin‐2
LGG: low‐grade glioma
LPS: lipopolysaccharide
MAPK: mitogen‐activated protein kinase
MCPIP: monocyte chemotactic protein‐induced protein
NF1: Neurofibromin 1
NF‐κB: nuclear factor kappa‐light‐chain‐enhancer of activated B cells
RBP: RNA‐binding protein
Reg‐2/Reg‐1: Regnase‐2/Regnase‐1
ROS: reactive oxygen species
SB: Sleeping Beauty transposon system
shRNA: short hairpin RNA
SL: stem‐loop structures
SOCS: suppressor of cytokine signaling
TGFβ: transforming growth factor beta
TIA‐1: T‐cell intracellular antigen 1
TIAR: TIA1‐related protein
TNBC: triple‐negative breast cancer
TNFα: tumor necrosis factor α
TRAF: tumor necrosis factor receptor–associated factor
TTP: tristetraprolin
UTR: untranslated region
VEGF: vascular endothelial growth factor
ZC3H12: zinc finger CCCH domain‐containing protein 12

## 1 INTRODUCTION

Persistent neuroinflammation is associated with neuroinflammatory and neurodegenerative diseases. Although there are no brain‐resident T or B cells in healthy individuals, peripheral immune cells cross the brain–blood barrier (BBB) under pathological conditions. Following the invasion of pathogens, brain cells including microglia, astrocytes, endothelial cells, oligodendrocytes, and neurons express inflammatory mediators that affect the integrity of the BBB and induce the recruitment of immune cells into the CNS.^1^ Moreover, the endogenous damage‐associated molecular patterns (DAMPs) also trigger neuroinflammation in the absence of pathogens as observed during CNS injury. Although the controlled release of DAMPs is beneficial for immune responses and tissue repair, it may lead to chronic neuroinflammation. Activation of pattern‐recognition receptors by DAMPs converges on several proinflammatory pathways, including the NF–κB and the MAPK pathways, which ultimately can lead to neurodegeneration.^2^ Sterile neuroinflammatory responses are associated with Alzheimer's disease, Parkinson's disease, Huntington's disease, amyotrophic lateral sclerosis, traumatic brain injury, stroke, and epilepsy.^3^, ^4^

In normal physiological conditions, the CNS undergoes constant immune surveillance primarily by the resident microglia (innate immune cells) and also astrocytes (non‐immune glial cells), which are crucial in the initiation, propagation, and regulation of neuroinflammation.^5^ Activation of microglia leads to their dramatic morphological and functional changes, acquisition of phagocytic phenotype, and production of proinflammatory molecules, including IL‐1β, IL‐6, TNFα, IL‐12, IL‐23, and reactive oxygen species (ROS).^6^, ^7^, ^8^ Similar to microglia, astrocytes become reactive, can proliferate, and synthesize ILs, TNFα, and ROS.^9^, ^10^

The physiological purpose of inflammation is to restore disturbed tissue homeostasis. However, unique immunosuppressive inflammation associated with glioblastoma multiforme (GBM) promotes tumor development, migration, invasion, proliferation, resistance to apoptosis, and maintenance of stem cell‐like properties.^11^, ^12^, ^13^, ^14^

Specific mechanisms controlling both the initiation and the resolution of inflammation are complex. Inflammation resolves through the scavenging of chemokines, cleaving of chemokines, switching the macrophage phenotype, inducing neutrophils apoptosis, and clearing neutrophils.^15^ Simultaneously, proinflammatory mediators induce the expression of anti‐inflammatory cytokines, such as IL‐10, IL‐4, IL‐13, TGFβ, and proteins that block pro‐inflammatory signaling, including SOCS and deubiquitinases.^16^, ^17^ The inhibition of proinflammatory responses also requires the degradation of the existing transcripts. Numerous RNA‐binding proteins (RBPs) and microRNAs interact with inflammatory mRNAs. RBPs recognize cis‐elements present in the 3′UTR, such as adenine‐ and uridine‐rich elements (AREs) or stem‐loop (SL) structures.^18^ AREs are the most well‐studied cis‐elements responsible for regulating the turn‐over of TNFα, GM‐CSF, IL‐3, IL‐2, IL‐6, IL‐8, iNOS, and COX‐2 mRNAs. These elements are bound by tristetraprolin (TTP), AUF‐1, KSRP, HuR, TIA‐1, and TIAR.^19^ Knock‐out of TTP, HuR, or AUF1 in mice triggers acute or chronic inflammation.^20^ Another set of RBPs, Roquin, Arid5a, and four proteins from the Regnase family (Reg‐1‐4) recognize SL structures with a pyrimidine‐purine‐pyrimidine loop sequence.^21^, ^22^, ^23^, ^24^, ^25^, ^26^, ^27^ Knock‐out of Roquin, *Reg‐1*, *Reg‐3*, and *Reg‐4* results in systemic inflammation in mice.^28^, ^29^, ^30^, ^31^ *Reg‐1*, the most studied member of the Regnase family, regulates inflammation via its RNase, deubiquitinase, and anti‐viral activities. *Reg‐1* degrades mRNAs encoding cytokines (IL‐6, IL‐1, IL‐12B, and IL‐2), chemokines (CXCL1, CXCL2, and CXCL3), T‐cell co‐stimulatory receptors (ICOS, TNFR2, and OX40), T‐cell activation marker (CD44), transcription factors (NFKBID, NFKBIZ, RelB, and IRF4), apoptotic factors (IER3), itself, and viral mRNAs.^22^, ^30^, ^32^, ^33^, ^34^, ^35^, ^36^ Reg‐1 also removes ubiquitin moieties from TRAF2, TRAF3, and TRAF6 thus negatively regulating JNK and NF–κB signaling pathways.^37^ Reg‐1 mRNA is highly abundant in immune tissues, spleen, and intestine. Although Reg‐1 levels are low in the brain, it has been postulated to function in the CNS.^38^, ^39^, ^40^, ^41^ In contrast, Reg‐2 functions remain elusive. We have shown that Reg‐2 regulates the half‐life of proinflammatory transcripts, such as IL‐6 mRNA.^26^ Since levels of Reg‐2 are high in the brain, we set up experiments to examine its role during neuroinflammation and glioblastoma progression.

## 2 MATERIALS AND METHODS

### 2.1 Cell culture

Primary human cortical astrocytes were prepared from fetal tissue provided by Advanced Bioscience Resources (Alameda, CA) as described previously.^42^ Astrocytes were initially cultured in DMEM supplemented with 10% fetal bovine serum, non‐essential amino acids, pen/strep, sodium pyruvate and glutamine. Astrocytes were serum starved for the experiments. The KMWT1 cell line is a mouse glioma established from the spontaneous glioma tumor. We have used CRE‐inducible oncogenic lentiviruses to generate it. These viruses expressing p53 shRNA and shRNA to NF1 induce tumors in Gfap‐Cre mice with histopathology and molecular signatures similar to human GBM. Tumors are induced using oncogenic lentiviruses in B6.Cg‐Tg(Gfap‐cre)77.6Mvs/2J driver mice that express the mouse Gfap promoter‐driven CRE in astrocytes and a subset of adult neural progenitor cells (inducing their differentiation to oligodendrocyte progenitor cells and their expansion). Spontaneous gliomas are generated within 6–9 weeks with 86% efficiency (*n* = 39) (Mockenhaupt et al, in revision). KMWT1 and U251‐MG cell lines were cultured in Dulbecco's modified Eagle's medium (DMEM) with 4.5 g/L glucose (Corning) supplemented with 10% tetracycline‐free fetal bovine serum (Biowest). The HeLa cell line was cultured in Dulbecco's modified Eagle's medium (DMEM) with 1.0 g/L D‐glucose (Lonza) supplemented with 2 mM L‐glutamine (Sigma‐Aldrich) and 10% fetal bovine serum (Biowest). Cell lines modified with the Sleeping Beauty transposon system were additionally supplemented with 1 μg/mL puromycin (Invitrogen). Cell lines were maintained at 37°C in a humidified atmosphere with 5% CO_2_. Cells were examined regularly for mycoplasma contamination using PCR.^43^

### 2.2 Cell stimulation

Cells were seeded in 12‐well plates. The following day the cells were pretreated with 1 μg/mL doxycycline for induction of transgene expression. 24 h later cells were prestimulated with cycloheximide (100 μg/mL, BioShop) for 1 h and/or stimulated with IL‐1β/TNF‐α (both 10 ng/mL, Invivogen). MG‐132 (1 μM, Merck) was added 1 h before IL‐1β stimulation.

### 2.3 Genetic constructs

Constructs for doxycycline‐inducible Regnase‐2 expression in the Sleeping Beauty transposon system (pSBtet‐GP‐Regnase‐2, pSBtet‐GP‐Regnase‐2‐D196A) were generated as described previously.^26^ The pSBtet‐Pur‐Clover‐Regnase‐2 vector used for live‐cell imaging was generated by standard cloning techniques. First, PCR products were obtained using pAZ0096‐CN7 (Clover) and pSB‐tet‐Pur‐SF‐Regnase‐2 templates with the PfuUltra II Fusion HS polymerase (Agilent), and the following primers: Sfi‐Clover‐Not‐for (5′‐AGGCCTCTGAGGCCCCTGCAGGCACCATGGTGAGCAAGGGCGAG‐3′), Sfi‐Clover‐Not‐rev (5′‐AGGCCTGACAGGCCGCGGCCGCGCCACCTCCGCTTCCACCTC‐3′), Not‐Regnase‐2‐Sfi‐for (5′‐AGGCCTCTGAGGCCGCGGCCGCAATGACGGCCACAGCTGAGGTAGAG‐3′), Not‐Regnase‐2‐Sfi‐rev (5′‐AGGCCTGACAGGCCGTTTAAACTCAACGTGCAGCCCTAAGCTTAG‐3′) introducing SfiI and NotI restriction sites. Next, the PCR products were digested using SfiI and NotI (New England Biolabs) and inserted into the pSBtet‐Pur vector linearized with SfiI using T4 DNA ligase (New England Biolabs).

The pSBtet‐GP‐3xTO‐Regnase‐2 vector was generated by replacing a fragment containing seven tet operators with a fragment containing three tet operators. 3xTO fragment was synthesized using the GeneArt Gene synthesis method (Invitrogen) and cloned into pSBtet‐GP vectors using standard cloning techniques.

The pLuc_*Reg‐2*_3′UTR construct encoding firefly luciferase transcript fused with the 3′UTR fragment of *Reg‐2* was generated by insertion of PCR amplified *Reg‐2* 3′UTR (NM_001010888.4) into the pmirGLO vector (Promega). Briefly, the *Reg‐2* 3′UTR fragment was amplified using cDNA obtained from U373‐MG cells with the Phusion™ Plus DNA Polymerase (Thermo Fisher Scientific), and the following primers: forward 5′‐GAGCTCCGTTGATATGACATAGTAC‐3′ and reverse 5′‐CTCGAGGGAAGGGCTATATTTTCACT‐3′. After agarose gel electrophoresis, the PCR product corresponding to the *Reg‐2* 3′UTR fragment was excised, purified, cleaved with SacI and XhoI (New England Biolabs), and inserted using T4 DNA ligase (New England Biolabs) into gel‐purified pmirGLO vector linearized with SacI and XhoI and dephosphorylated with CIP (New England Biolabs).

The plasmid encoding firefly luciferase transcript fused with 3′ truncations of *Reg‐2* 3′UTR was generated by restriction digestion using restriction sites naturally occurring within *Reg‐2* 3′UTR. The pLuc_*Reg‐2*_3′UTR plasmid was digested with SacI and EcoRI (pLuc_*Reg‐2*_3′UTR_1–1991) or XbaI (pLuc_*Reg‐2*_3′UTR_1–340) (New England Biolabs). The digestion products were resolved using agarose gel electrophoresis. The DNA bands corresponding to the truncated 3′UTR fragments were excised, purified, blunted with T4 DNA polymerase (New England Biolabs), and inserted using T4 DNA ligase (Thermo Fisher Scientific) into pmirGLO vector which was linearized with SacI and XhoI, previously blunted with T4 DNA polymerase and dephosphorylated with CIP (New England Biolabs).

The pLuc_*Reg‐1*_3′UTR construct encoding firefly luciferase transcript fused with the *Reg‐1* 3′UTR fragment was prepared as described previously.^44^ The expression plasmids for human Reg‐1, Reg‐1 RNase‐inactive mutant (D141A), Reg‐2, Reg‐2 RNase‐inactive mutant (D196A) were prepared as reported in Refs. [26, 44], respectively. The expression plasmid encoding human TTP was prepared by insertion of PCR‐amplified *ZFP36* (NM_003407.5) coding sequence (CDS) into the pcDNA3.1/mycHisA vector (Invitrogen). Briefly, The *ZFP36* CDS was amplified from the cDNA obtained from MCF‐7 cells with the Phusion™ Plus DNA Polymerase (Thermo Fisher Scientific) and the following primers: forward 5′‐GCTAGCATGGACTACAAAGACGATGAC‐3′ and reverse 5′‐CTCGAGTCACTCAGAAAACAGAGATGCG‐3′. After agarose gel electrophoresis, the PCR product corresponding to *ZFP36* CDS was excised, purified, cleaved with NheI and XhoI (New England Biolabs), and inserted using the T4 DNA ligase (Thermo Fisher Scientific) into gel‐purified pcDNA3.1/mycHisA vector linearized with NheI and XhoI and dephosphorylated with CIP (New England Biolabs). The expression vector coding for TTP RNase‐inactive mutant (C124R) was generated with the QuickChange II XL site‐directed mutagenesis kit (Agilent) using plasmid coding WT form as a template and the following primers: forward 5′‐TACGGGGCCAAGAGACAGTTTGCCCATGGCCTG‐3′ and reverse 5′‐CAGGCCATGGGCAAACTGTCTCTTGGCCCCGTA‐3′.

The expression plasmid for mouse Reg‐2 (pSBtet‐Pur‐Clover‐mReg‐2) was generated by replacing the human Reg‐2 CDS in pSBtet‐Pur‐Clover‐Regnase‐2 with PCR‐amplified mouse CDS (NM_001034907.3). The *Reg‐2* CDS was amplified from the cDNA prepared from MBE cells using the Q5 High Fidelity DNA Polymerase (New England Biolabs) and the following primers: mReg‐2‐NotI‐F: 5′‐AGCGGCCGCAATGACGGCCACAGCTGCAGTG‐3′ and mReg‐2‐PmeI‐R: 5′‐AGTTTAAACTCAACGTGCAGCTCTAAGCTTGGC‐3′. PCR product was cloned into a pJET1.2/blunt vector (Thermo Fisher Scientific) and then excised using NotI and PmeI restriction enzymes (New England Biolabs). After gel electrophoresis product corresponding to the mouse Reg‐2 CDS was purified. The pSBtet‐Pur‐Clover‐Regnase‐2 plasmid was digested using NotI and PmeI restriction enzymes. The digestion products were resolved using agarose gel electrophoresis and the DNA band corresponding to the vector backbone was excised, purified, and dephosphorylated with Fast AP Phosphatase (Thermo Fisher Scientific). Next, mouse *Reg‐2* CDS was ligated into the pSBtet‐Pur‐Clover backbone.

The expression plasmid encoding the mouse Reg‐2 with D195A mutation (pSBtet‐Pur‐Clover‐mReg2‐D195A) was prepared with the Q5 Site‐Directed Mutagenesis Kit (New England Biolabs) according to the manufacturer's protocol using pSBtet‐Pur‐Clover‐mReg‐2 vector as a template and following primers: mM2‐DAs: 5′‐AATTGTTATTgctGGAAGTAATGTG‐3′, mM2‐DAas: 5′‐GGTCTTAAATTATCACTATTGTC‐3′.

The pSBbi‐Pur‐dCas9‐KRAB‐meCP2‐hU6‐sgRNA‐SapI plasmid, used for Reg‐2 knockdown was a gift from Maria Czarnek.^45^ sgRNA sequence targeting *Zc3h12b* was designed using the CHOPCHOP tool.^46^ Oligonucleotides containing pSBbi‐Pur‐dCas9‐KRAB‐meCP2‐hU6‐sgRNA‐SapI compatible ends (mReg2_sg1_s: ACCGCCCCGCGACCACAGAACAG, mReg2_sg1_as: AACCTGTTCTGTGGTCGCGGGGC) were annealed and ligated into plasmid digested with SapI (New England Biolabs). All generated constructs were verified by Sanger sequencing (Genomed).

### 2.4 *Reg‐2* 3′UTR length determination

The length of *Reg‐2* 3′UTR was determined by the PCR analysis and 3′RACE (3′ Rapid Amplification of cDNAs Ends). Briefly, the total RNA was isolated from U251‐MG cells using the Chomczynski method.^47^ The cells were lysed with GTC buffer (4 M guanidinium thiocyanate, 25 nM sodium citrate, pH 7.0, 0.05% (w/v) sarkosyl, and 0.1 M 2‐mercaptoethanol) and total RNA was isolated using subsequent acid phenol‐chloroform extraction and isopropanol precipitation. RNA quality was assessed using standard agarose gel electrophoresis under denaturing conditions. RNA quantity was measured using a NanoDrop spectrophotometer (Thermo Fisher Scientific). For PCR analysis, cDNA was prepared by incubation of 1 μg of total RNA with M‐MLV reverse‐transcriptase (Promega) and oligo (dT)_15_ primer (Genomed) and proceed according to the manufacturer's instructions. Then the *Reg‐2* 3′UTR fragments were amplified from prepared cDNA using Phusion™ Plus DNA Polymerase (Thermo Fisher Scientific), and the following primers: forward 5′‐ GACAAAACCCAGGGGGAGAG‐3′ and a set of reverse primers R1: 5′‐ GCTGTGCACTGTCCTCCATT‐3′, R2: 5′‐TTGAGGCATTGCTCCTAGCC‐3′, R3: 5′‐TGGCCCTCAAAAAGGGCATT‐3′ and R4: 5′‐GACAAAACTGGGGAAGGGCT‐3′. The PCR products were separated on a 1% agarose gel. The 3′RACE was done using the SMARTer™ RACE cDNA kit (Clonetech, USA) according to the manufacturer's instructions. First cDNA was prepared from 1 μg of total RNA with primer 5′‐CCAGTGAGCAGAGTGACGAGGACTCGAGCTCAAGCTTTTTTTTTTTTTTTTT‐3′. Then two nested PCR were done with the following primers 1st RACE forward 5′‐CCACACAGCAGATGCC‐3′ and reverse 5′‐GAGGACTCGAGCTCAAGC‐3′, and 2nd RACE forward 5′‐CAGCAACTGGCAGCCTT‐3′ and 5′‐CCAGTGAGCAGAGTGACG‐3′. The RACE PCR products were separated on a 1% agarose gel. The results of electrophoresis were confirmed by the sequencing of the amplified bands.

### 2.5 Transfection, stimulation, and reporter gene assay

U251‐MG cells were seeded in 24‐well plates at a density of 1 × 10^5^ cells/well. 24 h later, the cells were transfected with PEI reagent (Thermo Fisher Scientific) according to the manufacturer's instructions. The total amount of 0.6 μg of plasmid DNA per well was used, including 0.4 μg of the luciferase‐coding reporter plasmid and 25 ng of Reg‐1, Reg‐2, TTP, corresponding RNase‐inactive mutant or the empty control plasmid. The quantity of DNA/well was normalized using an empty pcDNA3.1/mycHisA vector (Invitrogen). 24 h post‐transfection cells were stimulated with IL‐1β (Invivogen) or left untreated. 24 h after stimulation cells were lysed and firefly and *Renilla* luciferase activity were measured using Dual‐Luciferase Reporter Assay System (Promega), according to the manufacturer's instructions. For transfection efficiency normalization the *Renilla* luciferase, encoded on the same plasmid as the firefly luciferase (pmirGLO), was used. Luciferase data are presented as relative luciferase activity (firefly/*Renilla*) and normalized to 1.

### 2.6 Establishment of cell lines with inducible expression of Reg‐2

U251‐MG, HeLa or KMWT1 cells were seeded in a 12‐well plate and the following day were transfected using Lipofectamine 3000 (Thermo Fisher Scientific) or Lipofectamine LTX (Thermo Fisher Scientific) (for KMWT1 cells) with 900 ng of pSBtet‐GP, pSBtet‐GP‐Regnase‐2, pSBtet‐GP‐Regnase‐2‐D196A, pSBtet‐Pur‐Clover‐Regnase‐2, pSBtet‐Pur‐Clover‐mRegnase‐2, pSBtet‐Pur‐Clover‐mReg‐2‐D195A, pSBbi‐Pur‐dCas9‐KRAB‐meCP2‐hU6‐sgRNA‐SapI or pSBbi‐Pur‐dCas9‐KRAB‐meCP2‐hU6‐sgRNA‐SapI‐Reg‐2 and 100 ng of the pCMV(CAT)T7‐SB100 vector. 24 h later, cells were trypsinized and one‐fifth was transferred into 6‐well plates. Cells transfected with transposon vectors were selected using puromycin (1 μg/mL, Invivogen) for seven days. pSBtet‐GP was a gift from Eric Kovarz (Addgene plasmid # 60495)^48^ and pCMV (CAT)T7‐SB100 was a gift from Zsuzsanna Izsvak (Addgene plasmid #34879).^49^

### 2.7 Western blot analysis

Cells modified with SB transposons were seeded in 12‐well plates. 24 h after transfection protein expression was induced by doxycycline (1 μg/mL). Next day cells were lysed in SDS Loading Buffer (0.35 M Tris·HCl, 35% (v/v) glycerol, 10% (w/v) SDS, 3.6 M β‐mercaptoethanol, 0.12 g/mL bromophenol blue) and denatured in 95°C for 7 min. Protein extracts were analyzed by Western blot. Following antibodies were used: mouse monoclonal anti‐FLAG (F3165, Merck), anti‐HA‐Tag (C29F4, Cell Signaling Technology), anti‐Lamin B1 (D9V6H, Cell Signaling Technology), anti‐GAPDH (D16H11, Cell Signaling Technology), anti‐IκBα (L35A5, Cell Signaling Technology), anti‐β‐actin (8H10D10, Cell Signaling Technology), anti‐GFP (A‐11122, Invitrogen) anti‐mouseHRP (#7076), and anti‐rabbit HRP (#7074) (Cell Signaling). Luminescence was detected using the Clarity Western ECL Substrate (Bio‐Rad) and recorded using the Fusion‐Fx documentation system (Vilber Lourmat).

### 2.8 Reg‐2 dephosphorylation

Cells were lysed in lysis buffer (25 mM Tris pH 7.5, 100 mM NaCl, 1 mM DTT, 1% NP‐40) supplemented with the Halt protease inhibitor cocktail (Thermo Scientific), and incubated for 15 min on ice. Lysates were centrifuged (14 000 rpm, 10 min, 4°C), and the supernatant was collected. 40 μL of supernatant was mixed with the PMP buffer (New England Biolabs) and MnCl2 (1 mM), and treated with 400 U of λ‐phosphatase (New England Biolabs) for 60 min, 30°C. Then, SDS Loading Buffer (0.35 M Tris·HCl, 35% (v/v) glycerol, 10% (w/v) SDS, 3.6 M β‐mercaptoethanol, 0.12 g/mL bromophenol blue) was added and samples were denatured in 95°C for 7 min.

### 2.9 Immunoprecipitation

U251‐MG cells modified with pSBtet‐GP‐Regnase‐2 vector were seeded on 60‐mm cell culture dishes and the following day were transfected with a pRK5‐HA‐Ubiquitin‐WT plasmid encoding HA‐tagged Ubiquitin (pRK5‐HA‐Ubiquitin‐WT was a gift from Ted Dawson (Addgene plasmid # 17608))^50^ or left untreated. 4 h post‐transfection fresh medium with doxycycline (1 μg/mL) was added to induce transgene expression. The following day dishes were treated with IL‐1β (10 ng/mL) for 1 h or left untreated. Where indicated cells were pre‐treated with MG‐132 for 1 h before IL‐1β stimulation. Next, cells were washed twice with 1 mL of PBS and lysed in lysis buffer supplemented with phosphatase inhibitors (1× RIPA, 1× Halt Protease inhibitor cocktail (Thermo Scientific), 5 mM EDTA, 20 mM NaF, 100 mM β‐glycerophosphate, 20 mM Na_3_VO_4_, 1 mM MG‐132). Lysates were centrifuged for 15 min, 16 000 × *g* at 4°C. Supernatants were collected and FLAG‐Regnase‐2 was precipitated with 4 μg of anti‐FLAG antibody (F3165, Sigma‐Aldrich). The following day the antibody‐captured proteins were recovered by incubation with the Dynabeads Protein G (Thermo Fisher Scientific) magnetic beads (for 1 h at 4°C). After incubation, the beads were washed twice (5 min, RT) with lysis buffer. Finally, the beads were resuspended in the 5 times diluted SDS Loading Buffer (0.35 M Tris·HCl, 35% (v/v) glycerol, 10% (w/v) SDS, 3.6 M β‐mercaptoethanol, 0.12 g/mL bromophenol blue) and denatured at 95°C for 7 min.

### 2.10 Cell count/clonogenic assay

Clonogenic assay was performed as described previously.^45^ Briefly, cells were seeded in triplicates at a density of 100 cells per well on 6‐well plates. Doxycycline (1 μg/mL) was added daily to half of the wells to induce the expression of the transgene. When the visible colonies appeared (after ~7 days) cells were fixed using a clonogenic assay fixer/stain (6% (v/v) glutaraldehyde, 0.5% (w/v) crystal violet) and counted.

For cell count, assay KMWT1‐mReg‐2/mReg‐2 (D195A) and control (transfected with empty vector) cells were seeded in 12 well plates at the density of 20 000 cells per well. Doxycycline (1 μg/mL) was added every 24 h to induce transgene expression. The cells were trypsinized and counted after 24, 48, and 72 h. KMWT1 cells with constitutive knockdown of mReg‐2 (pSBbi‐Pur‐dCas9‐KRAB‐meCP2‐hU6‐sgRNA‐SapI‐Reg‐2) and cells modified with empty vector (pSBbi‐Pur‐dCas9‐KRAB‐meCP2‐hU6‐sgRNA‐SapI) were seeded in 6 well plates at the density of 50  000 cells per well. The cells were trypsinized and counted after 24, 48, and 72 h.

### 2.11 RNA isolation and reverse transcription

Before isolation tissue samples from patients were preserved in Nucleic Acid Preservation buffer, prepared according to Ref. [51], and stored at −80°C. Next, samples were homogenized in 1:1 phenol: GTC mixture (4 M guanidinium thiocyanate, 25 nM sodium citrate, pH 7.0, 0.05% (w/v) sarkosyl, and 0.1 M 2‐mercaptoethanol). Total RNA isolation was performed according to Chomczynski's modified protocol.^47^ The cells were lysed in a GTC buffer and RNA isolation was performed according to Chomczynski's protocol. RNA quantification and purity were assessed using spectrophotometric measurements using the NanoDrop ND‐1000 spectrophotometer (Thermo Fisher Scientific). RNA integrity was assessed by denaturing, formaldehyde gel electrophoresis. Reverse‐transcription reaction was performed using 1 μg of RNA with M‐MLV‐Reverse transcriptase (Promega) and 500 ng of oligo(dT) primers (or random hexamers for RNA isolated from patient's tissue samples) according to the manufacturer's instructions. The cDNA was used in quantitative real‐time PCR (qRT–PCR) for the evaluation of the amounts of the mRNAs of interest. The cDNA from the mouse brain was a gift from Maria Czarnek. Brains of C57BL/6J healthy, adult mice (6–8 weeks old) were obtained from the Animal Facility of the Faculty of Biochemistry, Biophysics and Biotechnology, Jagiellonian University, Kraków, Poland, pursuant to Directive 2010/63/EU of the European Parliament and of the Council.

### 2.12 qRT–PCR

The qRT–PCR was performed using RT‐HS‐PCR‐Mix‐SYBR‐A (A&A Biotechnology). Levels of the mRNAs of interest in each sample were analyzed in duplicates and the expression level was normalized to *EEF2 or TBP* and *RPL13A2* (for patient's samples). Expression levels were analyzed by the ΔΔCt method. Following primers were used: qRT–EEF2‐for (5′‐TACCTGGGAGAATCCACCGC‐3′), qRT‐EEF2‐rev (5′‐AGAAGAGGGAGATGGCAGTTGAC‐3′), qRT‐IL‐6‐for (5′‐GACAGCCACTCACCTCTTCA‐3′), qRT‐IL‐6‐rev (5′‐AGTGCCTCTTTGCTGCTTTC‐3′), qRT‐TBP‐for (5′‐GCCAGCTTCGGAGAGTTCTGGGATT‐3′), qRT‐TBP‐rev (5′‐CGGGCACGAAGTGCAATGGTCTTTA‐3′), qRT‐RPL13A2‐for (5′‐GA AGATGGCGGAGGTGCAG‐3′), qRT‐RPL13A2‐rev (5′‐CCTTCCGGCCCAGCA‐3′), qRT‐Reg‐2‐for (5′‐ATGTGGCAA TGAGCCATGGGAATA‐3′), qRT‐Reg‐2‐rev (5′‐TCATCATAGCAGACAACCCTCC‐3′), qRT‐TTP‐for (5′‐ACTTCAGCGCTCCCACTCTCGG‐3′), qRT‐TTP‐rev (5′‐CTCAGCGACAGGAGGCTCTCGTA‐3′), qRT‐Reg‐1‐for (5′‐GGAAGCAGCCGTGTCCCTATG‐3′), qRT‐Reg‐1‐rev (5′‐TCCAGGCTGCACTGCTCACTC‐3′), qRT‐Ef2‐for (5′‐CCACGGCAAGTCCACGCTGAC‐3′), qRT‐Ef2‐rev (5′‐AGAAGAGGGAGATGGCGGTGGATT‐3′), mReg‐2‐for (5′‐CATGGTCCGAGCCTTGAGAA‐3′), mReg‐2‐rev (5′‐CACTTGTGGCCGTAGGTACA‐3′), mIl‐6‐for (5′‐CAAAGCCAGAGTCCTTCAGA‐3′), mIl‐6‐rev (5′‐GATGGTCTTGGTCCTTAGCC‐3′). For mouse Reg‐1, commercially available primers were used (Bio‐Rad). Statistical analysis of qRT–PCR results was performed on the logarithm‐transformed relative quantification (RQ) values.^52^

### 2.13 Experimental autoimmune encephalomyelitis

Mice were immunized subcutaneously with 250 μg MOG^35^, ^36^, ^37^, ^38^, ^39^, ^40^, ^41^, ^42^, ^43^, ^44^, ^45^, ^46^, ^47^, ^48^, ^49^, ^50^, ^51^, ^52^, ^53^, ^54^, ^55^ peptide (AnaSpec, Fremont, CA) emulsified in CFA containing 500 μg *Mycobacterium tuberculosis* H37Ra (Difco, Detroit, MI). On days 0 and 3, each mouse was injected intraperitoneally with 200 ng of purified *Bordetella pertussis* toxin (Enzo Life Sciences, Farmingdale, NY). Mice were scored daily for the severity of the disease using a five‐point scale: 0, no symptoms; 1, limp tail; 2, limp tail with loss of righting; 3, paralysis of a single hind limp; 4, paralysis of both hind limps; and 5, moribund state or death. Mice were sacrificed on day 14 (peak symptomatic day). Spinal cord tissues were harvested and flash‐frozen and used for mRNA analysis.

### 2.14 LPS‐induced neuroinflammation

Mice were injected with PBS or lipopolysaccharide (5 μg/kg) by intraperitoneal injection. After 6 h, the animals were sacrificed to harvest brain tissues. The entire cerebrum was flash‐frozen, stored at −80°C, and used for mRNA analysis.

### 2.15 Isolation and culturing of primary cells from mice brains

The glial culture was harvested from P0‐P3 pups. After the removal of meninges, the whole brain was trypsinized for 30 min at 37°C and sieved through 100 μM nylon cell strainer. Cells were cultured in poly‐D‐lysine‐coated flasks in DMEM medium with 10% FBS. The media was replaced with fresh media every 48 h for a week.

For isolation of microglia, the mixed glial cell culture was shaken for 2 h at speed of 120 rpm at 37°C. The media that contains microglia was transferred to a new cell culture plate. Fresh DMEM media with 10% FBS was added to it. While the astrocytes that adhered to the flask after vigorous shaking were fed with 10% FBS for the next 2 days and plated for the experiment.

Both astrocytes and microglia were treated with LPS (100 ng/mL) or human IL‐1β (20 ng/mL).

### 2.16 Live‐cell imaging

Time‐lapse imaging was carried out using a 40× dry lens objective (HCX APO U‐V‐I 40×/0.75 DRY UV, Leica Microsystems) and a Leica DMi8 inverted fluorescence microscope (Leica Microsystems) equipped with Leica DFC7000 GT camera (Sony ICX674AL CCD, Leica Microsystems). During image acquisition, cells were maintained at 37°C in a humidified atmosphere with 5% CO_2_ (Pecon). Images were acquired as single *Z*‐planes. For quantitative and comparative imaging, identical image acquisition parameters were used for each set of experiments. The exact time‐lapse intervals and treatment conditions are indicated in the figure legends.

### 2.17 Meta‐analysis

Oncopression (http://www.oncopression.com/) database and GEPIA web server^53^ were used for the analysis of Reg‐1 and Reg‐2 expression levels between normal and cancer brain samples and their influence on glioma patient survival rates. To identify targeted Thr residues we have used bioinformatics tools such as platforms phosphosite,^54^ NetPhos3.1,^55^ PPSP (http://ppsp.biocuckoo.org/), GPS^56^ and MusiteDeep (www.musite.net/).

### 2.18 Statistical analysis

Statistical analysis was performed using Graph Pad Prism software (version 5.01, GraphPad Software Inc.). If not specified differently the asterisks denote the statistical significance of the indicated data point versus the control sample. The exact tests performed are indicated in the figure descriptions.

## 3 RESULTS

### 3.1 Coordinated regulation of Reg‐1 and Reg‐2 expression during neuroinflammation

**FIGURE 1:**
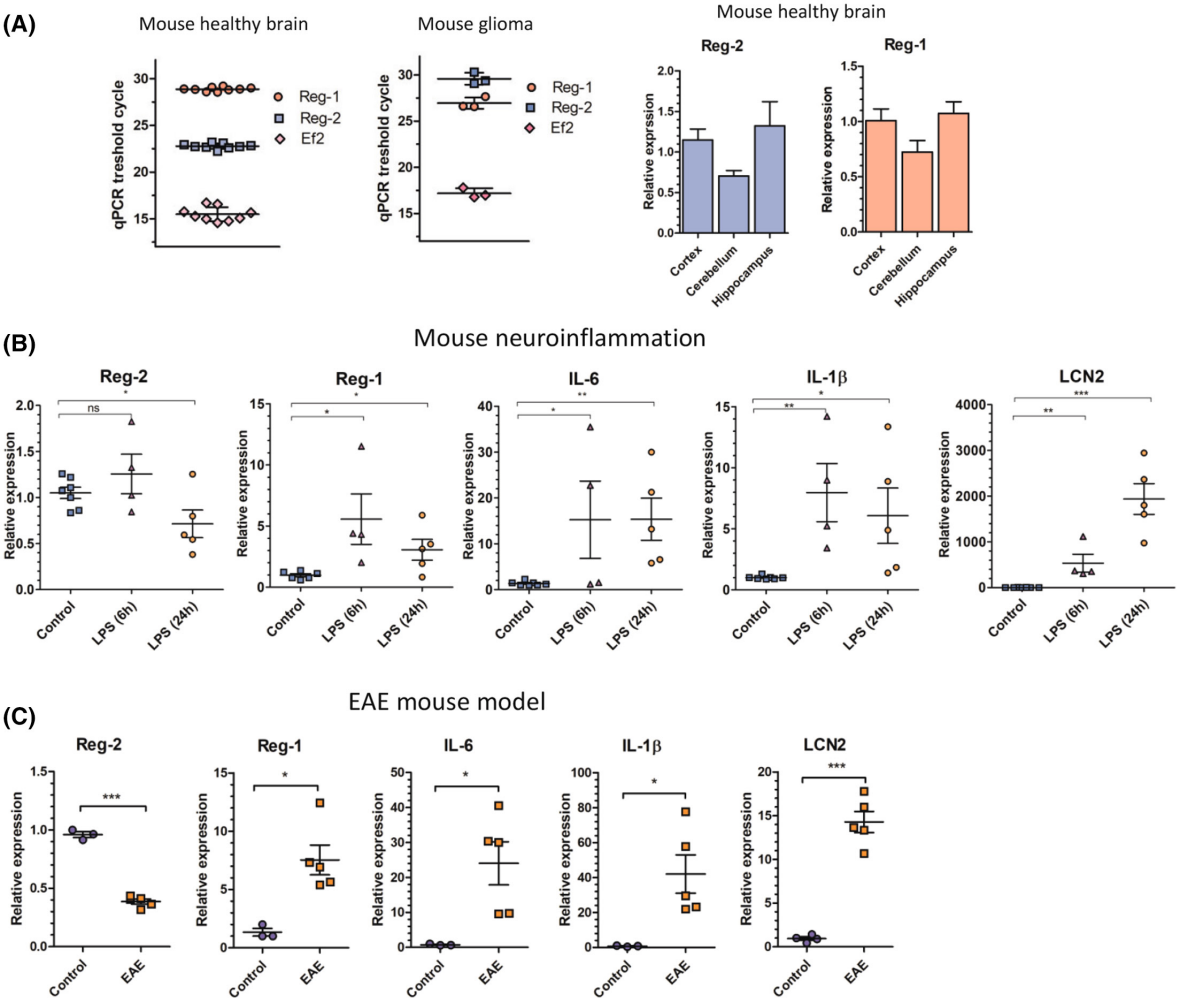
Coordinated regulation of Reg‐1 and Reg‐2 expression during neuroinflammation. (A) Comparison of the mRNA level of Reg‐2 and Reg‐1 in different mouse brain regions and spontaneous mouse glioblastoma. The Ct values are presented to illustrate the relative abundance of Reg‐2 and Reg‐1 (amplification efficiencies of all used primers were ~100%). Homogenized brain samples and mouse glioma cells were harvested and subjected to RNA isolation. The levels of the indicated mRNAs were examined using qRT–PCR. The graphs show the mean results of three independent experiments ±SD. (B) Regulation of Reg‐2 and Reg‐1 during LPS‐induced neuroinflammation in the mouse brain. The mRNA level of Reg‐2, Reg‐1, IL‐1β, IL‐6, and LCN2 in the mouse brain following LPS administration. Mice were injected intraperitoneally with PBS (control) or LPS (5 μg/kg). After 6 or 24 h, the animals were sacrificed to harvest brain tissues and the level of investigated transcripts was analyzed. (C) Regulation of Reg‐2 and Reg‐1 in the EAE mouse model. The mRNA level of Reg‐2, Reg‐1, IL‐1β, IL‐6, and LCN2 in the spinal cord of control and EAE mice. Mice were immunized subcutaneously with 250 μg MOG^35^, ^36^, ^37^, ^38^, ^39^, ^40^, ^41^, ^42^, ^43^, ^44^, ^45^, ^46^, ^47^, ^48^, ^49^, ^50^, ^51^, ^52^, ^53^, ^54^, ^55^ peptide emulsified in CFA containing 500 μg Mycobacterium tuberculosis H37Ra. On days 0 and 3, each mouse was injected intraperitoneally with 200 ng of purified Bordetella pertussis toxin. Mice were sacrificed on day 14 (peak symptomatic day). Spinal cord tissues were harvested and used for mRNA analysis. (B, C) Data are pooled from three independent experiments. The horizontal lines represent the mean ± SD. The data were analyzed using an unpaired *t*‐test. (*p < .05; **p < .01; ***p < .001).

We have previously reported that *Reg‐2* mRNA is expressed in the human brain and human cell lines of neural origin.^26^ Examination of a healthy mouse brain reveals that *Reg‐2* is highly expressed in the cortex, hippocampus, and cerebellum (Figure 1A). In contrast, *Reg‐1* is expressed at much lower levels in the healthy mouse brain (Figure 1A). Since *Reg‐2* mRNA is abundant in the healthy mouse brain, we wondered if its expression changes during experimental neuroinflammation. We have employed a mouse model of sterile neuroinflammation induced by peripheral administration of LPS.^57^ LPS induces systemic synthesis of cytokines, including TNFα, which cross BBB and trigger sterile neuroinflammation.^57^ TNFα activates microglia, astrocytes, and other cells in the CNS and increases the permeability of the BBB.^58^ As previously reported, LPS administration induced rapid expression of *IL‐1β* and *IL‐6* mRNA,^59^ and also a slower expression of *Lipocalin‐2* (*LCN2*) mRNA, which is the most induced transcript in the CNS^60^ (Figure 1B). *Reg‐1* mRNA levels were also rapidly increased while *Reg‐2* expression was decreased within 24 h (Figure 1B). To further investigate the regulation of Reg‐2 and Reg‐1 expression during neuroinflammation, experimental autoimmune encephalomyelitis (EAE) a mouse model of multiple sclerosis was used.^61^ Similar to what was found in the LPS‐induced model of neuroinflammation, *Reg‐2* mRNA levels were downregulated while expression of *Reg‐1*, *IL‐1β*, *IL‐6*, and *LCN2* mRNAs were upregulated in the spinal cords of the EAE mice (Figure 1C). These data suggest the existence of coordinated regulation of *Reg‐1/Reg‐2* expression with downregulation of *Reg‐2* expression and concomitant induction of *Reg‐1* expression during neuroinflammation.

### 3.2 Both astrocytes and microglia synthesize Reg‐2 and Reg‐1

**FIGURE 2:**
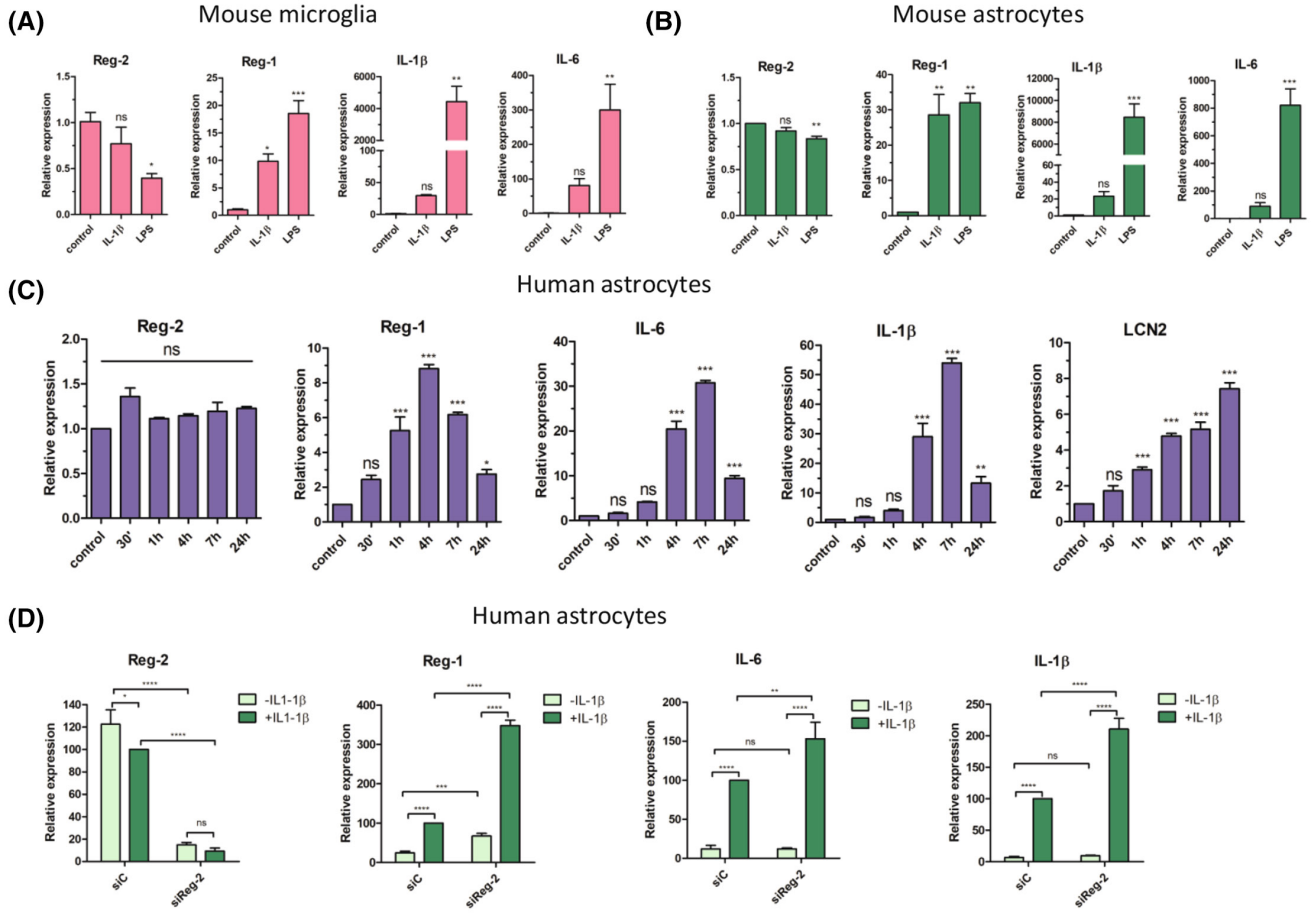
Regulation of Reg‐2 and Reg‐1 in (A) mouse primary microglial cells, (B) mouse primary astrocytes, and (C, D) human primary astrocytes. (D) cells treated with siRNA against Reg‐2 (siReg‐2) or scrambled siRNA (siC). (A–D) The cells were treated with LPS (100 ng/mL) (A, B) or human IL‐1β (20 ng/mL) (A–D) for 4 h or the indicated time, harvested and subjected to RNA isolation. The levels of the indicated mRNAs were examined using qRT–PCR. The graphs show the mean results of three independent experiments ±SD. The data were analyzed using one‐way ANOVA with Dunnet's post‐hoc test (*p < .05; **p < .01; ***p < .001).

To investigate the cellular and molecular mechanisms controlling coordinated *Reg‐1* and *Reg‐2* expression, we first concentrated on identifying cells expressing these two RNases. Both microglia and astrocytes are the main producers of cytokines in the CNS.^62^, ^63^ To mimic neuroinflammation primary mouse microglia and astrocytes were stimulated with LPS or IL‐1β. Stimulation of microglia with LPS resulted in downregulation of *Reg‐2* mRNA levels and upregulation of *Reg‐1* mRNA expression (Figure 2A). LPS also induced *Reg‐1* mRNA expression in astrocytes; however, *Reg‐2* mRNA levels were not affected in these cells (Figure 2B). *Reg‐1* mRNA expression was also induced by IL‐1β in both microglia and astrocytes, but *Reg‐2* mRNA levels remained unchanged. As expected, both LPS and IL‐1β strongly induced *IL‐1β* and *IL‐6* mRNA expression in both mouse microglia and astrocytes (Figure 2A,B). Subsequently, primary human astrocytes were stimulated only with IL‐1β since they do not respond to LPS.^64^ Similar to mouse astrocytes, *Reg‐2* mRNA expression was not affected by IL‐1β while *Reg‐1*, *IL‐1β*, and *IL‐6* mRNAs levels were increased following IL‐1β stimulation. While the expression of these mRNAs peaked at 4–7 h, the levels of *LCN2* mRNA gradually continued to increase (Figure 2C).

### 3.3 Reg‐2 regulates the level of *Reg‐1* and proinflammatory transcripts in primary human astrocytes

To examine the putative functions of Reg‐2, primary human astrocytes were transfected either with siRNA specific to *Reg‐2* (siReg‐2) or control siRNA (siC), and expression of *IL‐1β*, *IL‐6*, and *Reg‐1* mRNAs was analyzed. In unstimulated astrocytes, Reg‐2‐knock‐down significantly increased *Reg‐1* mRNA abundance but not *IL‐1β* and *IL‐6* transcripts (Figure 2D), suggesting that Reg‐2 controls homeostatic levels of Reg‐1. In IL‐1β‐stimulated astrocytes *IL‐1β*, *IL‐6*, and *Reg‐1* mRNAs expression was dramatically upregulated. Downregulation of *Reg‐2* in these cells significantly increased levels of all these transcripts (Figure 2D). Thus, Reg‐2 controls the turnover of proinflammatory cytokine transcripts as previously reported.^26^ However, Reg‐2 seems to possess a separate function regulating the turnover of *Reg‐1* mRNA, which encodes a cytokine‐inducible RNase.

### 3.4 Dysregulated *Reg‐2* and *Reg‐1* expression in glioblastoma multiforme

**FIGURE 3:**
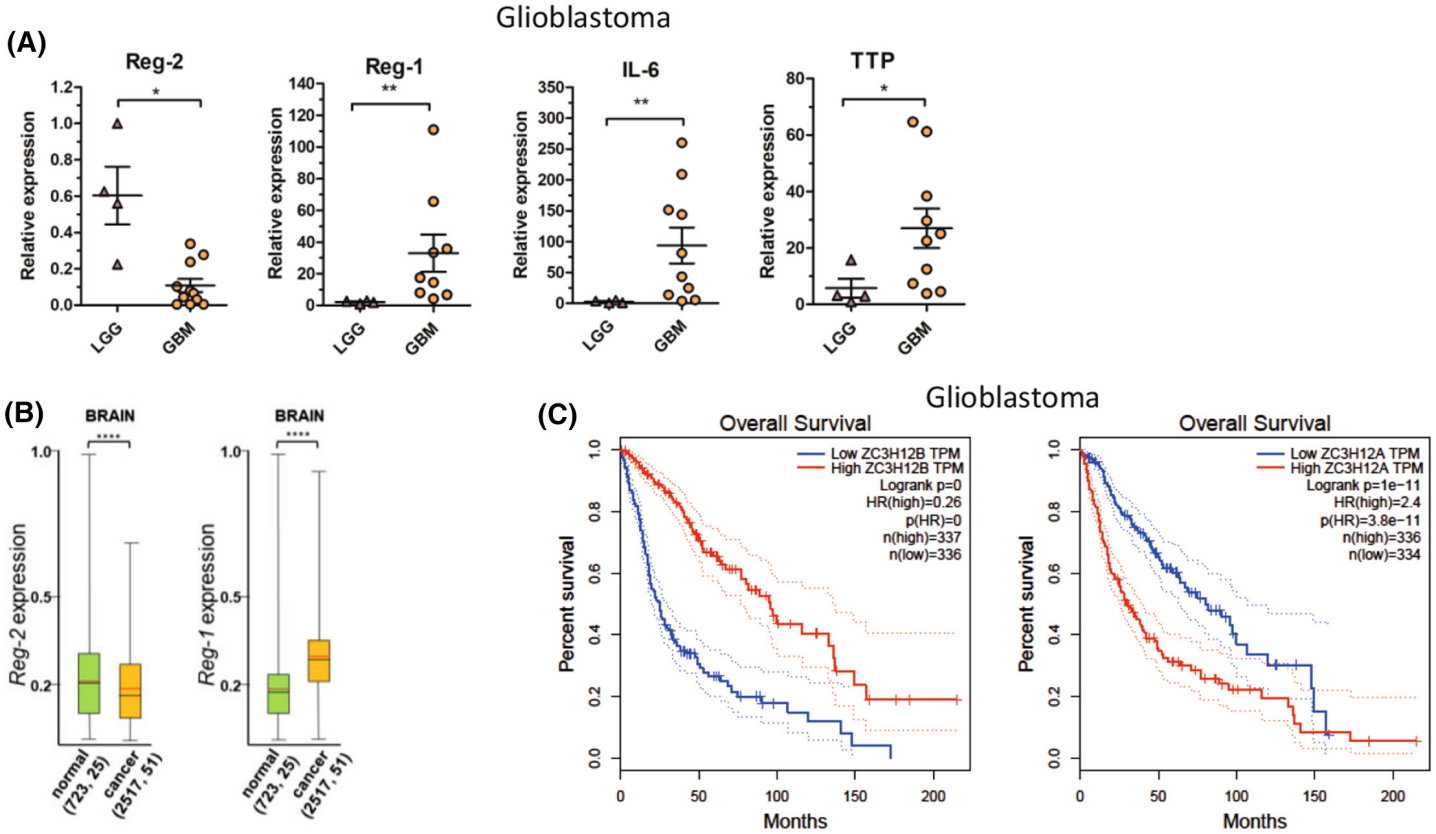
Regulation of Reg‐2 and Reg‐1 in glioblastoma samples. The mRNA level of Reg‐2, Reg‐1, IL‐6, and TTP in (A) clinical samples of low‐grade glioma (LGG) and glioblastoma multiforme (GBM)s. Samples from patients were homogenized and subjected to RNA isolation. Each point represents a single patient. The levels of the indicated mRNAs were examined using qRT–PCR. The horizontal lines represent the mean ± SD. The data were analyzed using the Mann–Whitney test. (*p < .05; **p < .01) (B) Expression of Reg‐2 and Reg‐1 in human brain normal and cancer tissues. Numbers in parentheses indicate the number of samples and data sets, respectively. The red line indicates the average, the black line the median, the box upper side the Q3 percentile and the lower side the Q1 percentile, and the upper and lower error bars indicate the maximum and minimum respectively. Graph prepared from the data obtained from the Oncopression database. (C) The prognostic relationship between Reg‐2 or Reg‐1 and patient survival. Graphs generated by GEPIA database.

We next examined the expression of *Reg‐2* and *Reg‐1* in a pathological setting using spontaneous mouse gliomas, human and mouse glioma cells, GBM patient samples, and deposited data. First, we examined spontaneous mouse gliomas (KMWT1 cell line), which we generated (Mockenhaupt et al, in revision) using CRE‐inducible oncogenic lentiviruses expressing p53 shRNA and NF1 shRNA.^65^ Surprisingly, our analysis revealed that *Reg‐2* and *Reg‐1* mRNA levels in spontaneous mouse gliomas dramatically differ from those in a healthy brain (Figure 1A). Whereas in a healthy brain *Reg‐2* mRNA is expressed at much higher levels than *Reg‐1* mRNA, *Reg‐2* mRNA expression levels are much lower than *Reg‐1* mRNA levels in mouse gliomas. These results indicated that the two RNases may play important roles during glioma development and progression. To further explore the regulation of *Reg‐1* and *Reg‐2* expression in pathological settings, we examined their expression in samples from patients with either low‐grade glioma (LGG) or GBM. In agreement with our findings in mouse gliomas, *Reg‐2* mRNA expression was significantly downregulated in GBM while *Reg‐1* mRNA expression was dramatically upregulated (Figure 3A). Patients with GBM were also characterized by enhanced expression of *IL‐6* and *TTP* mRNAs (Figure 3A). Furthermore, analysis of the *Oncopression* database indicated that *Reg‐2* mRNA levels are significantly downregulated in brain tumors in comparison to normal brain tissue, whereas *Reg‐1* mRNA levels are upregulated (Figure 3B). Significantly, low expression of *Reg‐2* and high expression of *Reg‐1* specifically predict shorter survival of GBM patients (Figure 3C). These data indicate that the regulation of expression levels of these two RNases may be important during tumorigenesis.

### 3.5 *Reg‐2* controls the expression of *Reg‐1* and inhibits cell proliferation

**FIGURE 4:**
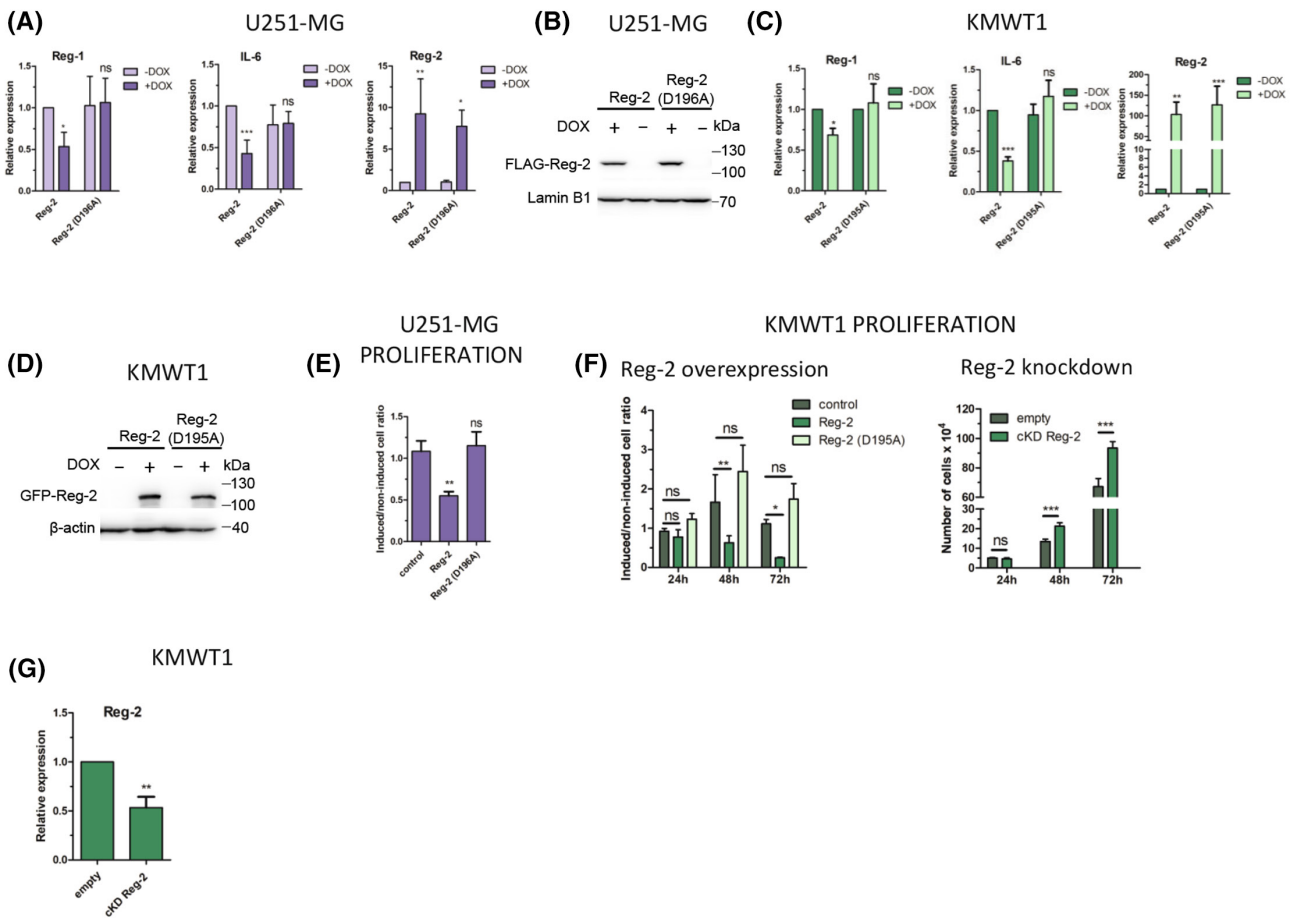
Reg‐2 regulates the level of Reg‐1 and IL‐6 in glioblastoma cells and inhibits glioblastoma cell proliferation in an NYN/PIN‐dependent manner. (A, B) Human glioblastoma U251‐MG (C, D) or mouse glioblastoma KMWT1 cells with Sleeping Beauty (SB) transposon‐based doxycycline (Dox)‐inducible expression of human or mouse wild‐type Reg‐2 or mutein Reg‐2 (D196A for human or D195A for mouse Reg‐2) were treated with Dox (1 μg/mL) or left untreated for 24 h, and the levels of the indicated mRNAs and proteins were examined using qRT–PCR and western blotting, respectively. (E) U251‐MG cells were plated on 6‐well plates (100 cells/well), and treated with Dox (1 μg/mL) daily, until visible colonies were formed (7 days). Then the colonies were counted. The data represents the ratio of colonies formed by Dox‐induced to noninduced cells normalized to the number of colonies formed by cells transfected with the empty control vector. (F) Mouse glioblastoma KMWT1 cells were treated with Dox (1 μg/mL) or left untreated and counted 24, 48, and 72 h after the addition of Dox. The data is presented as the ratios of the induced to the noninduced cells. Mouse glioblastoma cells, wild‐type or modified by CRISPRi system to down‐regulate the expression of Reg‐2 (cKD Reg‐2), were counted 24, 48, and 72 h after seeding. (G) Mouse glioblastoma KMWT1 cells were modified by CRISPRi system to downregulate the expression of Reg‐2. The level of Reg‐2 and Reg‐1 mRNA was analyzed by qPCR. The graphs show the mean results of three to six independent experiments ±SD. The data were analyzed using two‐way ANOVA and Bonferroni's post‐hoc test (****p < .0001; ***p < .001; **p < .01; *p < .05).

To examine whether Reg‐2 regulates *Reg‐1* mRNA expression, we have used both human U251‐MG glioblastoma cells and mouse KMWT1 glioma cell line established from the spontaneous glioma tumor (Mockenhaupt et al, in revision). Both cell lines were modified using the Sleeping Beauty transposon to enable the inducible expression of Reg‐2 or luciferase. Dox‐induced Reg‐2 expression downregulated *Reg‐1* mRNA levels in both cell lines (Figure 4A–D). This downregulation of *Reg‐1* mRNA expression required the NYN/PIN domain of Reg‐2, since expression of either human Reg‐2 (D196A) or mouse Reg‐2 (D195A) inactive mutants did not affect *Reg‐1* mRNA levels (Figure 4A–D). We concluded that Reg‐2 actively controls the levels of *Reg‐1* mRNA. The downregulation of Reg‐2 expression in glioma cells may be required for their proliferation as Reg‐2 inhibits the proliferation of Flp‐In T‐REx‐293 cells.^26^ Indeed, the proliferation of both human U251‐MG and mouse KMWT1 cells was inhibited by the wild‐type Reg‐2 but not mutant Reg‐2 (Figure 4E,F). Conversely, CRISPRi‐mediated knockdown of Reg‐2 (Figure 4G) accelerated the proliferation of the KMWT1 cells (Figure 4F).

### 3.6 IL‐1β‐induces post‐translational modifications of Reg‐2

**FIGURE 5:**
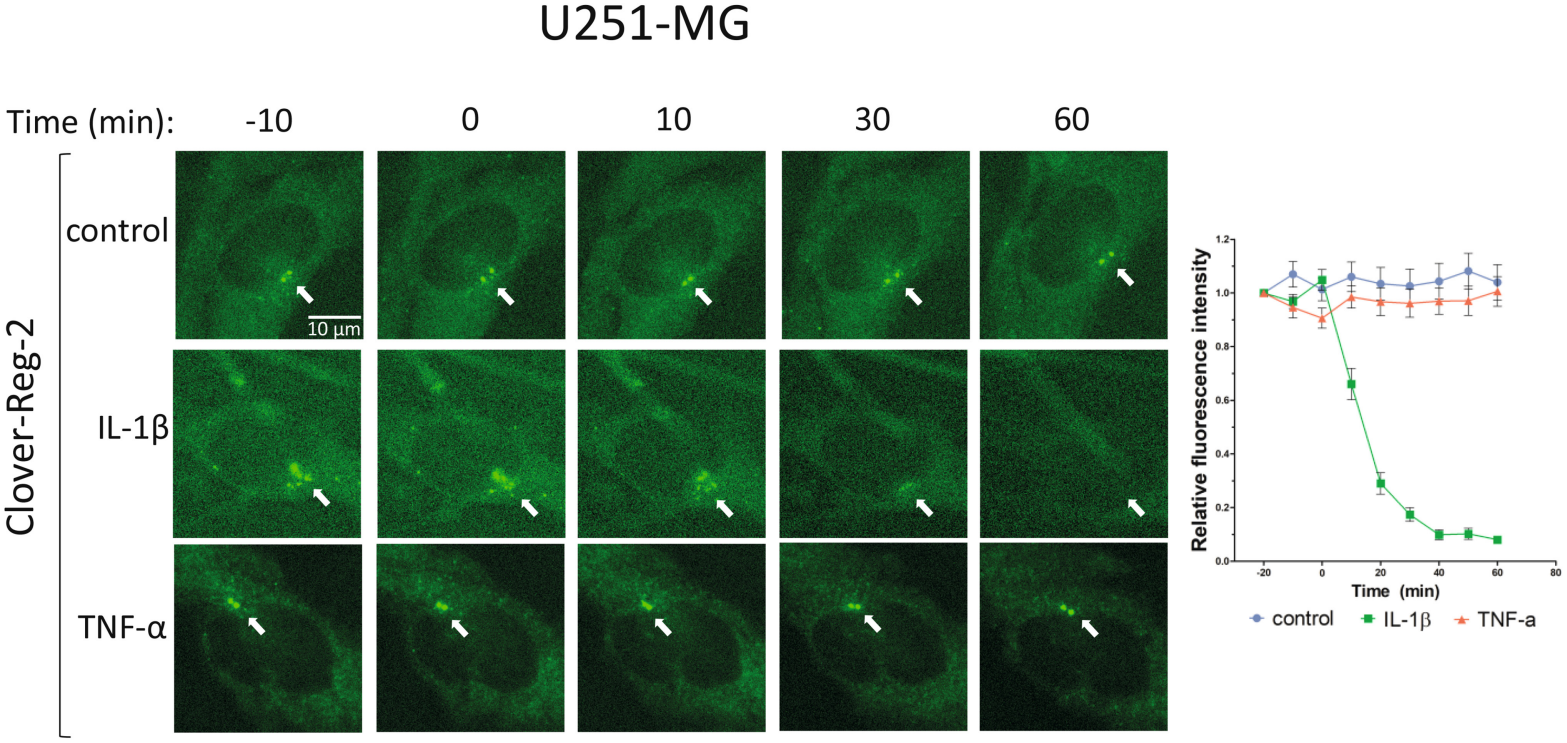
Live‐cell imaging of Reg‐2 decay following IL‐1β stimulation. U251‐MG cells with SB transposon‐based Dox‐inducible expression of Clover‐tagged wild‐type Reg‐2 were treated with Dox (1 μg/mL) for 24 h and then stimulated with IL‐1β (10 ng/mL) or TNFα (10 ng/mL) or left unstimulated. Clover‐Reg‐2 was visualized in living cells using a widefield fluorescence microscope. Image analysis was performed using Image‐J software. Each data point represents the amount of wild‐type Reg‐2 in untreated cells (control, blue circles) or cells stimulated with IL‐1β (green squares) or TNFα (red triangles) for the indicated time. Data points: average normalized fluorescence intensity measured from at least 20 random cells from three independent experiments ±SEM.

**FIGURE 6:**
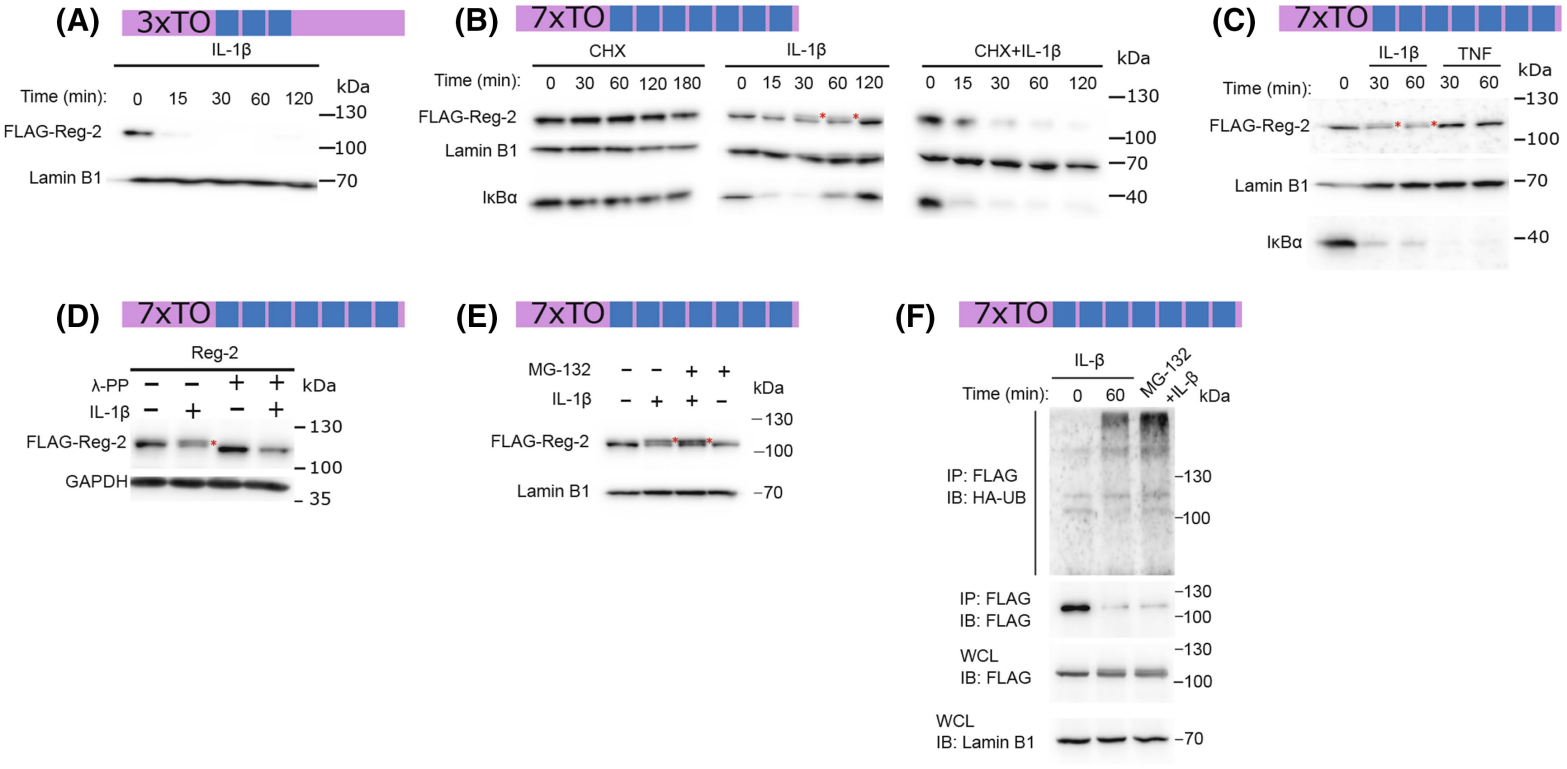
IL‐1β induces modification and degradation of Reg‐2. U251‐MG cells with SB transposon‐based Dox‐inducible expression of FLAG‐tagged wild‐type Reg‐2 under the control of a promoter containing three tetracycline operator sites (3xTO; A) or seven tetracycline operator sites (7xTO; B–F) were treated with Dox (1 μg/mL) for 24 h. Then, 7xTO cells were pre‐treated with cycloheximide (CHX) (100 μg/mL) for 1 h or left untreated and stimulated with IL‐β (10 ng/mL) or TNF (10 ng/mL) (B, C) while 3xTO cells were stimulated with IL‐1β only (A). (D) The cells were treated with Dox (1 μg/mL) for 24 h and stimulated with IL‐1β (10 ng/mL) for 60 min. The protein lysate was incubated with phosphatase λ‐PP (60 min, 37°C). (E) The modification of Reg‐2 following IL‐β stimulation (10 ng/mL, 60 min) with an MG‐132 proteasome inhibitor (1 μM) was examined. (F) Immunoassay of lysates of U251‐MG cells with Dox‐inducible expression of FLAG‐tagged wild‐type Reg‐2 under the control of 7xTO promoter and transfected with the expression vector encoding HA‐tagged Ubiquitin. The cells were stimulated for 0–30 min with IL‐1β (10 ng/mL), followed by Reg‐2 immunoprecipitation (IP) and immunoblot analysis (IB) with antibody to ubiquitin (Ub) or Reg‐2 (FLAG). Levels of the indicated proteins were examined by the western blotting. Red asterisks indicate a slower migrating band. Data are representative of three to five independent experiments.

Since *Reg‐2* mRNA levels are downregulated during inflammation and development of glioma, we subsequently analyzed the expression of Reg‐2 protein. We generated U251‐MG cells with inducible expression of Clover‐Reg‐2 fusion protein and examined its levels in cells stimulated with IL‐1β and TNFα using fluorescent microscopy. We observed a very rapid decrease of Clover‐Reg‐2 protein levels with a half‐life of less than 15 min upon IL‐1β stimulation (Figure 5), which suggested an active IL‐1β‐induced decay of the protein. In contrast, stimulation with TNFα did not affect Clover‐Reg‐2 protein levels (Figure 5). IL‐1β also induced rapid decay of Clover‐Reg2 in HeLa cells (Figure S1), suggesting a universal mechanism controlling Reg‐2 protein abundance. To further study Reg‐2 protein regulation, we examined control and IL‐1β‐treated U251MG‐Reg‐2 cells. Although Reg‐2 protein was expressed by control cells, its levels rapidly decreased following IL‐1β stimulation (Figure 6A). To study the possible mechanisms, we generated cells expressing high levels of Reg‐2 using the strong 7xTO promoter, which allowed us to investigate possible posttranslational modifications. Using this experimental setup, the IL‐1β‐induced decay of Reg‐2 was especially evident in the presence of cycloheximide (CHX), which blocks the synthesis of new proteins (Figure 6B). However, we detected a slower migrating band, which was rapidly induced by IL‐1β (Figure 6B, middle panel, IL‐1β) but not TNFα (Figure 6C). The slower migrating IL‐1β‐induced band suggested that Reg‐2 may be phosphorylated. To verify this hypothesis, samples were treated with phosphatase. The slower migrating bands were sensitive to phosphatase suggesting that IL‐1β stimulation induces phosphorylation of Reg‐2 (Figure 6D).

Phosphorylation of proteins and their ubiquitination often leads to their degradation.^66^ We have employed the proteasome inhibitor MG‐132 and found that pretreatment of cells with MG‐132 results in the accumulation of the IL‐1β‐induced slower migrating band (Figure 6E). IL‐1β also induced ubiquitination of Reg‐2, which was particularly evident once protein degradation was inhibited by MG‐132 (Figure 6F).

### 3.7 The control of Reg‐2 transcript turnover

**FIGURE 7:**
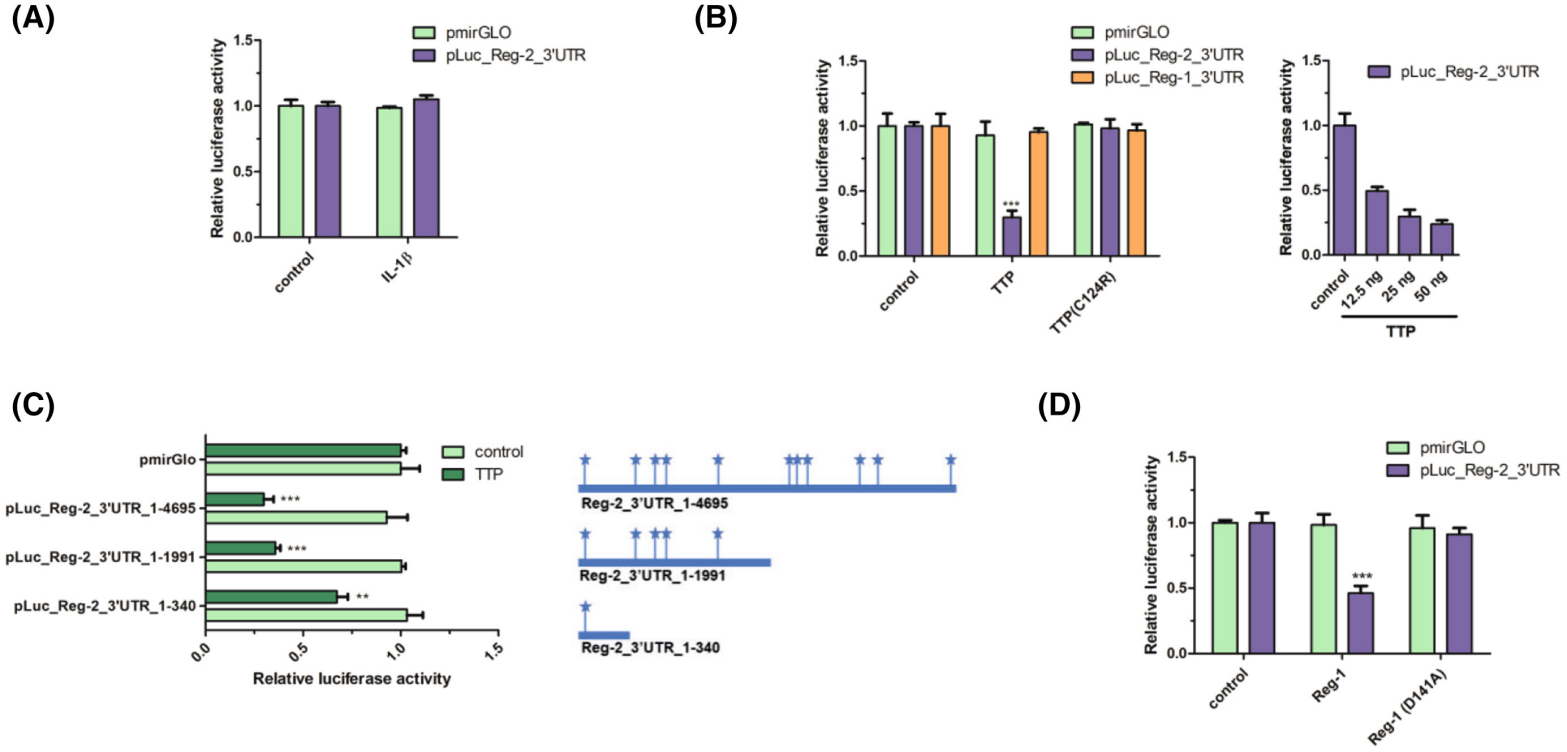
TTP and Reg‐1 are involved in the 3′UTR‐dependent destabilization of Reg‐2 transcripts. (A) U251‐MG cells were transfected with vectors encoding luciferase with the attached 3′UTR of wild‐type Reg‐2 or without an additional 3′UTR (pmirGLO). Where indicated the cells were stimulated with IL‐1β (10 ng/mL). (B) U251‐MG cells were transfected with vectors encoding luciferase with the attached 3′UTR of wild‐type Reg‐2 or 3′UTR of wild‐type Reg‐1 or without any additional 3′UTRs (pmirGLO) and an expression vector for TTP (25 ng) or inactive mutein (C124R) (25 ng) or an empty control vector (pcDNA3.1 (left panel). U251‐MG cells were transfected with vectors encoding luciferase with attached Reg‐2 3′UTR (pLuc_Reg‐2_3′UTR) and varying amounts (12,5, 25, and 50 ng/well) of TTP expression vectors or an empty control vector (pcDNA3.1) (right panel). (C) U251‐MG cells were transfected with vectors encoding luciferase with truncation mutants of Reg‐2_3′UTR or without additional 3′UTR (pmirGLO) and TTP expression vectors (25 ng) or an empty control vector (pcDNA3.1). The diagram represents the localization of ARE sequences in the Reg‐2 3′UTR and their contents in generated truncated mutants of Reg‐2 3′UTR. (D) U251‐MG cells were transfected with vectors encoding luciferase with the attached 3′UTR of wild‐type Reg‐2 or without additional 3′UTR (pmirGLO) and an expression vector for wild‐type Reg‐1 or RNase inactive mutein Reg‐1(D141A) or an empty control vector (pcDNA3.1). The graphs show the mean results of three to four independent experiments ±SD. The data were analyzed using one‐way ANOVA (A,C,D) or two‐way‐ANOVA (B) and Bonferroni's post‐hoc test (*p < .05; **p < .01; ***p < .001).

Our data suggest that Reg‐2 levels are controlled at both mRNA and protein levels. We subsequently examined *Reg‐2* transcript stability. First, we verified that the *Reg‐2* mRNA indeed contains a 4695 bp‐long 3′UTR, which is much longer than an average 3′UTR of 800 bp^67^ and much longer than the *Reg‐2* coding sequence. The *Reg‐2* 3′UTR is also significantly longer than the *Reg‐1* 3′UTR, which is only 766 bp‐long. The Reg‐2 3′UTR was verified to be 4695 bp‐long using PCR and 3′RACE (3′ Rapid Amplification of cDNAs Ends) (Figure S2). Second, we cloned the *Reg‐2* 3′UTR into the luciferase reporter and used this construct in the transfection experiments. We did not find any changes in the luciferase activity in cells treated with IL‐1β (Figure 7A), which corroborated our findings in astrocytes and microglia (Figure 2A–C). Third, we performed the *Reg‐2* 3′UTR sequence analysis that identified numerous ARE sequences (AU‐rich elements) and potential alternative polyadenylation sequences (Figure 7C). Since tristetraprolin (TTP) is one of the best‐known regulators interacting with ARE sequences,^68^, ^69^ we co‐transfected the pLuc_Reg2_3′UTR reporter and either wild‐type (wt) TTP or its inactive C124R mutant.^70^ As a negative control, pLUC_Reg1_3′UTR luciferase reporter containing wt *Reg‐1* 3′UTR lacking AREs was used. Expression of the wt TTP but not its inactive C124R mutant dramatically reduced the activity of the pLuc_Reg2_3′UTR reporter, but had no effect on the control pLUC_Reg1_3′UTR reporter (Figure 7B), suggesting specific regulation of *Reg‐2* mRNA stability by TTP. Importantly, the effect of TPP on the pLuc_Reg2_3′UTR reporter was TTP dose‐dependent, indicating high specificity (Figure 7B). Analysis of the luciferase reporters, containing truncated *Reg2*_3′UTR, demonstrated that all the ARE sequences contribute to the TTP‐mediated control (Figure 7C). Fourth, since TTP negatively regulates *Reg‐2* mRNA levels and low *Reg‐2* mRNA expression predicts short GBM patients' survival (Figure 3C), we examined whether high levels of TTP can be found in GBM samples. Indeed, levels of *TTP* are much higher in tumors from patients with GBM than LGG (Figure 3A). Fifth, we examined whether Reg‐1 regulates the abundance of *Reg‐2* mRNAs using the pLuc‐Reg2‐3′UTR reporter in transfection experiments. We found that wt Reg‐1 but not its inactive D141A mutant^30^ significantly diminished reporter activity (Figure 7D), indicating that Reg‐1 regulates the half‐live of *Reg‐2* mRNA. Thus, these two RNases, Reg‐1 and Reg‐2, negatively control the abundance of each other's mRNAs, which likely fine tunes responses during inflammation as well as tumorigenesis.

## 4 DISCUSSION

The proteins from the Regnases/ZC3H12/MCPIP family were discovered more than 10 years ago, but their role during both physiological and pathological processes is still not fully understood. All of these proteins possess the critical nucleolytic NYN/PIN domain responsible for transcripts degradation. Reg‐1, ‐3 and ‐4 are also important modulators of several cell signaling pathways regulating immune and inflammatory responses.^29^, ^37^, ^71^ However, our understanding of the biological functions of *Reg‐2* is scarce.^26^, ^72^ Recently, we have shown that *Reg‐2* regulates *IL‐6* mRNA levels in an NYN/PIN‐dependent manner. *Reg‐2* also inhibits the proliferation of Flp‐In T‐REx‐293 cells by stalling the cell cycle in the G2 phase.^26^

In this report, we demonstrate that *Reg‐2* is highly expressed in all regions of the healthy mouse brains while *Reg‐1* levels are very low. However, *Reg‐2* levels are dramatically downregulated during both EAE‐associated and LPS‐induced neuroinflammation as well as immunosuppressive neuroinflammation associated with mouse gliomas. In contrast, levels of *Reg‐1* are dramatically induced in these neuroinflammatory conditions. Importantly, *Reg‐2* expression also negatively correlates with the grade of glioma in clinical samples of patients, and low expression of *Reg‐2* predicts short survival. Our current data suggest that *Reg‐2* regulates the expression of several proinflammatory cytokines and restricts the proliferation of human and mouse glioma cells. Since extensive proliferation and development of unique immunosuppressive inflammation are hallmarks of human GBM,^73^ our results suggest that *Reg‐2* negatively regulates these critical processes. Thus, downregulation of *Reg‐2* expression may be required for the development of immunosuppressive neuroinflammation, GBM development and progression.

Interestingly, *Reg‐2* levels negatively correlated with the levels of *Reg‐1*. Reg‐2 destabilizes *Reg‐1* transcripts while Reg‐1 destabilizes *Reg‐2* transcripts, and these effects depend on the NYN/PIN domains of these two RNases. This intricate regulation seems to be needed for the fine‐tuning of the inflammatory responses. The final ratio of both RNases is crucial for GBM patients' prognosis with low levels of Reg‐2 and high levels of Reg‐1 correlated with poor survival. We have also found that *Reg‐2* mRNA turnover is regulated by TTP, and the levels of TTP are strongly increased in GBM. Collectively, our data suggest that both Reg‐1 and TTP expression is upregulated but *Reg‐2* mRNA levels is downregulated in high‐grade gliomas. Our current findings in mouse and human glioma cells are unexpected. Both Reg‐1 and Reg‐2 have been described as inhibitors of cellular growth.^26^, ^74^, ^75^ Reg‐1 has been shown to inhibit the proliferation of Caki‐1 cells by downregulating *DDB1* mRNA, which in turn increases the p21Cip1 levels.^76^ Although Reg‐2 downregulates *DDB1* transcript levels in Flp‐In T‐REx‐293 cells, the p21Cip1 levels are also decreased in these cells.^26^ Interestingly, overexpression of Reg‐1 arrests cells in the G0/G1 phase whereas Reg‐2 overexpression stalls the cells in the G2 phase of the cell cycle.^26^, ^74^, ^77^ Thus, although both RNases require intact NYN/PIN domain to regulate cell proliferation their targets must be different.

In addition, the levels of Reg‐1 decrease during the progression of clear cell renal cell carcinoma with low Reg‐1 levels correlating with increased proliferation, vascularity, and tumor outgrowth. This has been associated with decreased Reg‐1 RNase activity, increased secretion of VEGF, IL‐8, CXCL12, and phosphorylation of VE‐cadherin.^75^ Reg‐1 has also been described as a potent antioncogene in breast cancer involved in the degradation of anti‐apoptotic transcripts and suppression of TGF‐β signaling.^78^, ^79^ Overexpression of Reg‐1 leads to inhibition of proliferation of triple‐negative breast cancer cells (TNBC) both in vitro and in vivo.^74^ Thus, the strong upregulation of Reg‐1 observed in high‐grade glioblastoma and the correlation of high‐Reg‐1 expression with poor patient prognosis is unexpected.

Our results demonstrate that Reg‐2 expression is downregulated during the development of neuroinflammation. Mechanistically, this downregulation occurs on both mRNA and protein levels. Reg‐1 and TPP seem to be critical for the decay of *Reg‐2* mRNA. We also demonstrate that Reg‐2 protein is phosphorylated, ubiquitinated, and degraded by the proteasome following IL‐1β stimulation. In contrast, treatment of cells with TNF does not result in Reg‐2 degradation. These results suggest that the molecules activated by IL‐1 receptor, but not TNF receptor, such as MyD88 and IRAKs may be required to induce posttranslational modifications of Reg‐2.

Taken together, our analysis of human GBM samples, spontaneous mouse gliomas, and mouse models of neuroinflammation conclusively demonstrate Reg‐2 downregulation. While there are no reports on the regulation of the expression of *Reg‐2* transcription, we have revealed the mechanisms regulating Reg‐2 expression on the posttranscriptional and posttranslational levels. Presented data indicate that TTP and Reg‐1 participate in *Reg‐2* mRNA turnover. In turn, phosphorylation of Reg‐2 leads to its ubiquitination and degradation following IL‐1β treatment. Our data also suggest that Reg‐2 plays homeostatic roles in normal physiological conditions restricting cell proliferation and inflammation in the brain. Further studies are needed to fully understand the homeostatic role of Reg‐2.

## 5 CONCLUSION

Overall, our data demonstrate that Reg‐2 restricts proliferation and expression of proinflammatory cytokines in resting astrocytes thus regulating brain homeostasis. Reg‐2 expression is downregulated during neuroinflammation and glioblastoma progression. We identify both posttranscriptional and posttranslational mechanisms that control Reg‐2 expression.

## AUTHOR CONTRIBUTIONS

Conceptualization, Aneta Kasza, Tomasz Kordula, Mateusz Wawro, and Weronika Sowinska; Investigation, Weronika Sowinska, Mateusz Wawro, Debolina D. Biswas, Jakub Kochan, Katarzyna Pustelny, Aleksandra Solecka, Angela S. Gupta, Karli Mockenhaupt, and Aneta Kasza; Methodology, Mateusz Wawro, Weronika Sowinska, Jakub Kochan, and Debolina D. Biswas; Jarosław Polak, and Borys Kwinta contributed glioblastoma samples; Supervision, Aneta Kasza, Tomasz Kordula, and Mateusz Wawro; Writing original draft, Weronika Sowinska, Aneta Kasza, and Tomasz Kordula.

## DISCLOSURES

The authors declare no conflict of interest.

## ETHICAL APPROVAL AND CONSENT TO PARTICIPATE

The Jagiellonian University Ethics Committee approval for human glioblastoma samples collection and analysis #1072.6120.65.2020. Mice were housed at Virginia Commonwealth University according to the Institutional Animal Care and Use Committee guidelines. Animals were housed with a 12‐h light/dark cycle, in an animal facility with standard laboratory chow, and water ad libitum. Randomly chosen littermates (males and females) were used for all experiments.

## CONSENT FOR PUBLICATION

All the authors have read and agreed to the published version of the manuscript.

## Supporting information


Text S1‐S2

Figure S1‐S2

## Data Availability

All data supporting the conclusions of this article are included within the article and its supplemental file. Information regarding the experimental methods used, and the data in this paper are available to scientific communities upon direct contact to the authors.
