# Supplementary material for: The homeostatic function of Regnase‐2 restricts neuroinflammation
Source: FASEB J. 2023 Feb 8;37(3):e22798. doi: 10.1096/fj.202201978R (PMC9983307; doi:10.1096/fj.202201978R)
Supplement: Supplementary file 1 — Text S1‐S2 [file FSB2-37-0-s001.docx]

**SUPPLEMENTARY FIGURE LEGENDS**

***Fig. S1. Live-cell imaging of Reg-2 decay following IL-1β stimulation.*** *HeLa cells with SB transposon-based Dox-inducible expression of Clover-tagged wild-type Reg-2 or mutein Reg-2 (S703,705,707A) were treated with Dox (1 μg/mL) for 24 hrs and then stimulated with IL-1β (10 ng/ml) or left unstimulated. Clover-Reg-2 was visualized in living cells using a widefield fluorescence microscope.*

***Fig. S2. Reg-2 transcript possesses an extraordinarily long 3’UTR region****.* ***(A)*** *Bioinformatic analysis of the 3’UTR region of Reg-2 transcript reveals the presence of numerous regulatory ARE sequences (AU-rich elements) and the potential alternative polyadenylation sequences. 3’UTR sequence of Reg-2 was analyzed with AREsite2.0 software (*[*http://rna.tbi.univie.ac.at/AREsite2*](http://rna.tbi.univie.ac.at/AREsite2)*). The length of 3’UTR was verified by PCR analysis* ***(B)*** *and 3’RACE* ***(C)****. The cDNA was generated by reverse transcription of RNA isolated from U251-MG. Then the cDNA was analyzed either by PCR with a single forward primer and a set of reverse primers (schematically depicted), or by 3’RACE with two nested PCR (1st RACE and 2nd RACE). Optimization of the reaction conditions (temperature and concentration of primers) eliminated non-specific reaction products. The PCR products were run on a 1% agarose gel.*
