# Supplementary figures and images for: The homeostatic function of Regnase‐2 restricts neuroinflammation
Source: FASEB J. 2023 Feb 8;37(3):e22798. doi: 10.1096/fj.202201978R (PMC9983307; doi:10.1096/fj.202201978R)

S1

HeLa

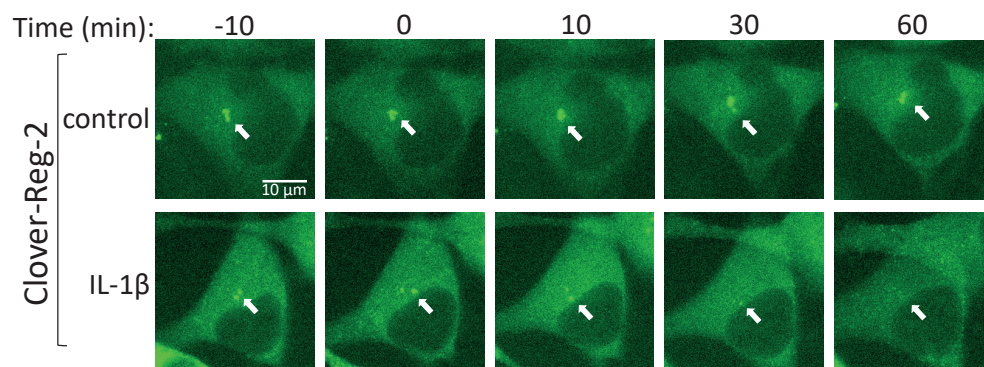

S2

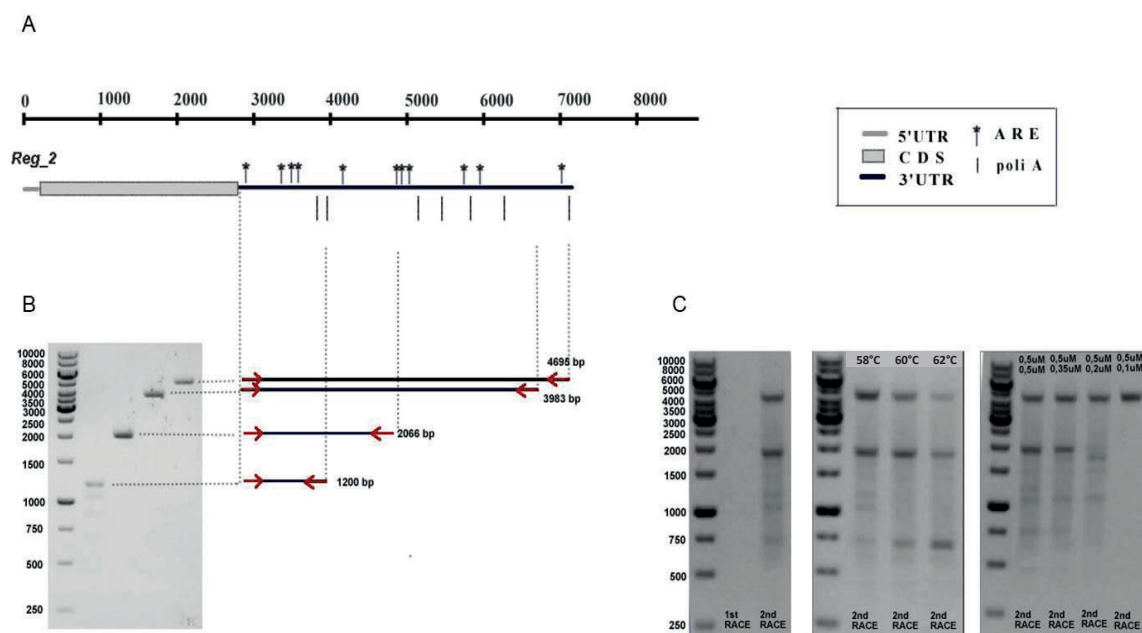

Supplement: Supplementary file 2 — Figure S1‐S2 [file FSB2-37-0-s002.pdf]
